# Finite-Key Analysis for Quantum Key Distribution with Discrete-Phase Randomization

**DOI:** 10.3390/e25020258

**Published:** 2023-01-31

**Authors:** Rui-Qiang Wang, Zhen-Qiang Yin, Xiao-Hang Jin, Rong Wang, Shuang Wang, Wei Chen, Guang-Can Guo, Zheng-Fu Han

**Affiliations:** 1 CAS Key Laboratory of Quantum Information, University of Science and Technology of China, Hefei 230026, China; 2 CAS Center for Excellence in Quantum Information and Quantum Physics, University of Science and Technology of China, Hefei 230026, China; 3 Hefei National Laboratory, University of Science and Technology of China, Hefei 230088, China; 4 State Key Laboratory of Cryptology, Beijing 100878, China; 5 Department of Physics, University of Hong Kong, Pokfulam, Hong Kong

**Keywords:** quantum key distribution, finite-key analysis, discrete-phase randomization

## Abstract

Quantum key distribution (QKD) allows two remote parties to share information-theoretic secret keys. Many QKD protocols assume the phase of encoding state can be continuous randomized from 0 to 2π, which, however, may be questionable in the experiment. This is particularly the case in the recently proposed twin-field (TF) QKD, which has received a lot of attention since it can increase the key rate significantly and even beat some theoretical rate-loss limits. As an intuitive solution, one may introduce discrete-phase randomization instead of continuous randomization. However, a security proof for a QKD protocol with discrete-phase randomization in the finite-key region is still missing. Here, we develop a technique based on conjugate measurement and quantum state distinguishment to analyze the security in this case. Our results show that TF-QKD with a reasonable number of discrete random phases, e.g., 8 phases from {0,π/4,π/2,…,7π/4}, can achieve satisfactory performance. On the other hand, we find the finite-size effects become more notable than before, which implies that more pulses should be emit in this case. More importantly, as a the first proof for TF-QKD with discrete-phase randomization in the finite-key region, our method is also applicable in other QKD protocols.

## 1. Introduction

Quantum key distribution (QKD) [1,2],one of the most successful and mature applications in quantum information science, allows for two legitimate parties (Alice and Bob) to share information-theoretic secret keys. In theory, its security has been proved [3,4,5], while experiments towards a higher key rate [6] and longer achievable distance [7,8,9,10] have been demonstrated. Still, some large scale QKD networks are emerging [11,12,13,14]. However, owing to the inherent photon-loss in the channel, it meets a vital bottleneck that limits the communication distance and key generation rate. Specifically, some fundamental rate-loss limits [15,16] impose a restriction on any point-to-point QKD without repeaters. More precisely, the key rate *R* is bounded by the channel transmission probability η with the linear PLOB bound $R=-\log_{2}\left(1-\eta \right)$ [16]. Delightfully, M.Lucamarini et al. made a breakthrough by proposing twin-field (TF) QKD in 2018. The essential idea of TF-QKD is in code mode extracting the key bit from a single-photon click event of the measurement station located in the middle of channel, which happens with a probability proportional to $\sqrt{\eta }$; thus, surpassing the linear PLOB bound becomes possible, and a so-called phase-error rate may be estimated in decoy mode [17,18,19] to monitor security. Driven by this, several TF-type QKD protocols [20,21,22,23,24,25,26] were proposed later to complete security proofs and improve performance. Based on these protocols, experimentalists also made great efforts to realize TF-QKD [27,28,29,30,31,32,33,34,35].

Since TF-QKD inherits measurement-device-independent (MDI)-QKD’s [36] merit that is immune to all side-channel attacks to measurement devices and all measurement-device imperfections [37,38], one does not need to take the detection loopholes into account within the TF-type QKD system. In spite of this, the security issues of the state preparation in TF-QKD must be carefully considered. In practice, the laser source of TF-QKD is usually a continuous source emitting coherent states with a fixed phase. Meanwhile, continuous phase-randomization from 0 to π is required in the TF-QKD. More specifically, this continuous phase-randomization is assumed in both the code and test modes in Refs. [21,23,26], or at least in the test mode in Refs. [22,24,39]. To fulfill this requirement, Alice and Bob must randomize the global phase continuously and uniformly. Unluckily, two ways to achieve phase randomization introduce different problems in the experiment. Passive randomization will lead to phase correlations between adjacent pulses [40,41], while active randomization can only randomize the phase over discrete set of values.

To bridge this gap between theory and experiment, two works that analyzed the security of fully discrete-phase randomization TF-QKD protocol have been proposed [42,43,44] in these days. However, a security proof in the finite-key region is still missing. Hence, one natural question is that whether TF-QKD with fully discrete-phase randomization can work well non-asymptotically. This work affirms that it can.

In this paper, we analyze the security of TF-QKD protocol with fully discrete randomization in a finite-key region. Interestingly, our analysis leads to comparable performance with the continuous one. Since taking the discrete phase into account, our results make the TF-QKD more practical and can be applied to the future TF-QKD experiment. More importantly, some techniques proposed here, e.g., Lemma A1 (introduced later), can be utilized to analyze the security of other QKD protocols with discrete-phase randomization.

This work is organized as follows. In Section 2, we give a description of the TF-QKD protocol with fully discrete-phase randomization, and the sketch of the security proof is given in Section 3. Note that the proof is detailed in Appendix A. In Section 4, by the numerical simulation, we show this protocol can still beat the linear PLOB bound [16] and has satisfactory performance. Finally, a conclusion is given in Section 5.

## 2. Protocol Description

Indeed, the protocol analyzed here has been depicted in Ref. [43]. For ease of understanding, we illustrate the protocol as follows.

Step 1: Alice (Bob) chooses a label from {“μ”,“0”,“ν”} with probabilities $P_{\mu },P_{O},P_{\nu }$, according to the label she (he) chooses, she (he) takes one of the following actions:

“μ”: she (he) randomly picks an integer $l_{A_{c}}$ ($l_{B_{c}}$) from {0,1,⋯,M−1} with equal probability $\frac{1}{M}$ where M is an even integer. This means that the phase 2π is divided into M parts. Then, she (he) randomly chooses a key bit $k_{a}$$\left(k_{b}\right)$ where $k_{a}\left(k_{b}\right)\in \left\{0,1\right\}$. Finally, she (he) sends a pulse with a coherent state $|e^{i(\frac{l_{Ac}}{M}2\pi +\pi k_{a})}\sqrt{\mu }\rangle$($|e^{i(\frac{l_{B_{c}}}{M}2\pi +\pi k_{b})}\sqrt{\mu }\rangle$ ).

“0”: she (he) sends the vacumm state.

“ν”: she (he) randomly picks an integer $l_{A_{c}}$ and $l_{B_{c}}$ from {0,1,⋯,M−1} with equal probability $\frac{1}{M}$ where M is an even integer. This means that the phase 2π is divided into M parts. Then, she (he) sends a pulse with a coherent state $|e^{i\frac{l_{Ac}}{M}2\pi }\sqrt{\mu }\rangle$($|e^{i\frac{l_{Bc}}{M}2\pi }\sqrt{\mu }\rangle$ ).

The first case is called code mode, while the other cases are decoy mode.

Step 2: Alice and Bob repeat Step 1 in total of $N_{tot}$ times.

Step 3: After receiving $N_{tot}$ pairs of pulses from Alice and Bob, interfering each pair at a beamsplitter and measuring the two outputs with his single photon detectors (SPDs), an honest Eve announces whether or not each measurement is successful. Here, ’successful’ means only one SPD (left SPD or right SPD) clicks in the corresponding measurement, and if so, Eve reports the specific SPD clicked.

Step 4: For those rounds Eve announcing successful click, Alice and Bob announce the intensities they chose as well as the values of $l_{A_{c}}$ and $l_{B_{c}}$. Then, Alice and Bob only retain those successful rounds in which the intensities of the coherent state they sent are same while the in-phase ($l_{A_{c}}=l_{B_{c}}$) or anti-phase ($|l_{A_{c}}-l_{B_{c}}|=M/2$) condition is also met. Let $n_{2\beta }^{+}$($n_{2\beta }^{-}$) be the number of the retained rounds when both Alice and Bob chose the same intensity β of the coherent state and the in-phase (anti-phase) is also met. Note that we assume $l_{A_{c}}=l_{B_{c}}=0$ always holds in the case of β=0. Alice and Bob generate their sifted keys from $n_{2\mu }=n_{2\mu }^{+}+n_{2\mu }^{-}$ retained rounds in code mode, thus the length of sifted key bits $n_{bit}=n_{2\mu }$. Note that if it is an in-phase (anti-phase) round with right (left) SPD clicking, Bob may flip his corresponding sifted key bit.

Step 5: With all of the quantities $n_{2\beta }=n_{2\beta }^{+}+n_{2\beta }^{-}$, Alice and Bob use linear programming to obtain an upper bound on the number of phase errors(defined later) $n_{ph}^{U}$ with a failure probability no more than ε; then, they can calculate the upper bound $e_{ph}^{U}=n_{ph}^{U}/n_{bit}$.

Step 6: Step 6 consists of error correction and privacy amplification.

Step 6a: Alice sends $H_{EC}$ bits of syndrome information of her sifted key bits to Bob through an authenticated public channel. Then, Bob uses it to correct errors in his sifted keys. Alice and Bob calculate a hash of their error-corrected keys with a random universal hash function and check whether they are equal. If equal, they continue to the next step; otherwise, they abort the protocol.

Step 6b: Alice and Bob apply the privacy amplification to obtain their final secret keys. If the length of their secret key satisfies $l=n_{bit}\left(1-h\left(e_{ph}^{U}\right)\right)-H_{EC}-\log_{2}\frac{2}{\epsilon_{cor}}-\log_{2}\frac{1}{4\epsilon_{PA}^{2}}$ where h(·) denotes the binary Shannon entropy, this protocol must be $\epsilon_{cor}$-correct and $\epsilon_{sec}$-secret with $\epsilon_{sec}=\sqrt{\varepsilon }+\epsilon_{PA}$. Here, $\epsilon_{cor}$($\epsilon_{sec}$) represents the protocol is correct (secret) with a failure probability no more than $\epsilon_{cor}\left(\epsilon_{sec}\right)$. Hence, the total security parameter is $\epsilon_{tol}$-secure where $\epsilon_{tol}=\epsilon_{cor}+\epsilon_{sec}$. It is elaborated thoroughly in the widely-used universally composable security framework [45,46].

## 3. Security Proof

In this section, we present the security proof of this protocol. The main task of the security proof is to bound the information Eve holds. To accomplish this task, one can calculate a so-called phase-error rate. Firstly, we construct an equivalent virtual protocol, in which Alice and Bob prepare some entangled states between local states and traveling states, but traveling states must have the same density matrices as actual protocol in the channel. The sifted key bits can be seen as the outputs of measurement with *Z*-basis on local states made by Alice and Bob; then, the so-called phase-error rate is defined as the error rate for the outputs of measurement with the *X*-basis made by them. According to the complementarity argument [47], the phase-error rate can be used to bound Eve’s information on the sifted keys. In the following, we give the virtual protocol and show how to bound the phase-error rate.

### 3.1. Equivalent Virtual Protocol

In our virtual protocol, Alice generates secret keys from the code mode in which she prepares the state

(1) $$\begin{array}{c} {|\psi \rangle }_{\mu ,A_{c}Aa}=\sum_{l=0}^{M-1}\frac{1}{\sqrt{M}}{|l\rangle }_{A_{c}}(\frac{1}{\sqrt{2}}{(|0\rangle }_{A}|e^{i\frac{2\pi }{M}l}\sqrt{\mu }\rangle_{a}+{|1\rangle }_{A}|-e^{i\frac{2\pi }{M}l}\sqrt{\mu }\rangle_{a})), \end{array}$$

where $A_{c}$ and A are the local quantum systems in Alice’s side, and *a* is the traveling quantum state Alice sent to Eve. Similarly, Bob prepares ${|\psi \rangle }_{\mu ,B_{c}Bb}$ defined analogously to ${|\psi \rangle }_{\mu ,A_{c}Aa}$. Obviously, Alice (Bob) measures A(B) with *Z*-basis to obtain sifted key, i.e., ${|0\rangle }_{A}$ for bit 0 and ${|1\rangle }_{A}$ for bit 1. In order to obtain the phase-error rate, they measure A,B in *X*-basis {|+〉,|−〉} after Eve’s attack. As for the test mode, we assume Alice prepares the following states

(2) $$\begin{array}{cc} {|\psi \rangle }_{0,A_{c}Aa} & ={|0\rangle }_{A_{c}}{|0\rangle }_{A}{|0\rangle }_{a},\\ {|\psi \rangle }_{\nu ,A_{c}Aa} & =\sum_{l=0}^{M-1}\frac{1}{\sqrt{M}}{|l\rangle }_{A_{c}}{|0\rangle }_{A}|e^{i\frac{2\pi }{M}l}\sqrt{\nu }\rangle_{a}. \end{array}$$

here, the local states of $A_{c}$ are encoded in photon-number states, and Alice can measure $A_{c}$’s photon-number to learn the phase of sent states.

Finally, we can describe the process of state preparation above with a single state, namely,

(3) $$\begin{array}{c} {|\psi \rangle }_{A_{s}A_{c}Aa}=\sqrt{p_{\mu }}{|0\rangle }_{A_{s}}{|\psi \rangle }_{\mu ,A_{c}Aa}+\sqrt{p_{O}}{|1\rangle }_{A_{s}}{|\psi \rangle }_{0,A_{c}Aa}+\sqrt{p_{\nu }}{|2\rangle }_{A_{s}}{|\psi \rangle }_{\nu ,A_{c}Aa} \end{array}$$

where Alice’s additional local ancilla $A_{s}$ is in the photon number states. Similarly, Bob can prepare ${|\psi \rangle }_{B_{s}B_{c}Bb}$ defined analogously to ${|\psi \rangle }_{A_{s}A_{c}Aa}$. Though Alice (Bob) may measure $A_{s}$($B_{s}$),$A_{c}\left(B_{c}\right)$, and *A*(*B*) after or before Eve announcing her measurement results, Alice (Bob) must announce the measurement results after Eve’s announcement then post-select the successful rounds. The following is a detailed illustration of our equivalent virtual protocol.

Step 1:

Alice and Bob prepare a gigantic quantum state $|\Phi \rangle ={|\phi \rangle }^{⊗N_{tot}}={(|\psi \rangle }_{A_{s}A_{c}Aa}⊗{|\psi \rangle }_{B_{s}B_{c}Bb}{)}^{⊗N_{tot}}$ and send all subsystems *a* and *b* to Eve through an insecure quantum channel.

Step 2:

After performing an arbitrary quantum operation on all subsystems *a* and *b* from Alice and Bob, Eve announces whether it has a successful click (only one of her SPDs clicks) or not for each round. For a successful round, Eve continues to announce whether the left SPD clicks or the right SPD clicks. We use M($\bar{M}$) to denote the set of successful (unsuccessful) rounds.

Step 3:

For those rounds in which Eve announces success, Alice and Bob jointly measure the subsystem $A_{c}$($B_{c}$) and $A_{s}\left(B_{s}\right)$ in the photon-number basis to learn whether the intensities of the coherent state they send are same or not and whether it is in-phase or anti-phase. Then, they only retain those rounds where in-phase or anti-phase is met, and they choose the same intensities. Let $M_{s}$ denote the set of those retained rounds, while $M_{f}$ denotes those rounds that are in M but not in $M_{s}$.

Step 4:

For these rounds in $M_{s}$, Alice (Bob) measures the subsystem $A_{c}A_{s}$($B_{c}B_{s}$) in Fock basis to learn the phase and intensity of the coherent states she (he) sent. If the result of $A_{s}$($B_{s}$) is in state ${|0\rangle }_{A_{s}}$(${|0\rangle }_{B_{s}}$), she (he) measures subsystems *A*(*B*) in the Z basis to decide her (his) sifted key, respectively; otherwise, she (he) measures subsystem A(B) in the Z basis but does not incorporate these measurement outcomes in her (his) sifted key.

Step 5 to Step 6:

Let $n_{2\beta }$ be the number of rounds in $M_{s}$ satisfying that both Alice and Bob chose the intensity β. With parameters $n_{2\beta }$, perform the same operations as Step 5 to Step 6, respectively, in the actual protocol given in Section 2.

### 3.2. Estimation of Phase-Error Rate

The essential of security proof is to estimate the upper-bound of the phase-error rate $e_{ph}$ of the sifted keys, i.e., how many same or different outcomes Alice and Bob have if they measure *A* and *B* with *X*-basis hypothetically in the rounds where sifted keys are generated. Specifically, in our protocol, we define the number of the same outcomes they have as $n_{ph}$, i.e., the number of phase-error events. Provided that $e_{ph}=n_{ph}/n_{2\mu }$ is bounded, one can generate the final secret key with an appropriate $\epsilon_{tol}$ value as given in Step6.b of the actual protocol.

A detailed proof for how to estimate $n_{ph}$ is present in Appendix A. Here, a sketch of this proof is given.

Though analyzing the equivalent protocol, it is proven that if Alice and Bob both chose intensity β, and in-phase or anti-phase is also met, they actually prepare a mixture $\tau_{2\beta }$, which consists of component $\tau_{j|2\beta },j=0,1,...,M-1$. Moreover, each phase-error event is a click by some particular components of that mixture $\tau_{2\beta }$, i.e., $\tau_{j|2\beta },j=0,2,...,M-2$. These results imply that

(4) $$\begin{array}{cc} n_{2\mu }= & \sum_{j=0}^{M}n_{j|2\mu },\\ n_{2\nu }= & \sum_{j=0}^{M}n_{j|2\nu },\\ n_{ph}= & \sum_{j=0,j\in N_{0}}^{M-2}n_{j|2\mu }, \end{array}$$

where $n_{j|2\beta }$ denotes the number of rounds in $M_{s}$, in which Alice and Bob both chose intensity β, but $\tau_{2\beta }$ is actually $\tau_{j|2\beta }$. Meanwhile, $N_{0}$ is the set of even numbers. Now, the hypothetical value $n_{ph}$ is related to some experimentally observed values. However, just with these equations it is difficult to bound $n_{ph}$ tightly since $n_{j|2\beta }$ cannot be known directly.

On the other hand, both $\tau_{j|2\mu }$ and $\tau_{j|2\nu }$ are very close to Fock-state |j〉〈j|. Accordingly, it is intuitive to consider if there are constraints on the gap between $n_{j|2\mu }$ and $n_{j|2\nu }$. Then, we developed Lemma A1 (see Appendix A for details) to bound the gap between the yields of two distinct quantum states in a non-asymptotic situation. Applying this lemma, we obtained a series of constraints on $n_{j|2\mu }$ and $n_{j|2\nu }$. Finally, combined with Equation (4), an analytical upper bound of $n_{ph}$ (given in the end of the Appendix A) was calculated to find the upper bound of phase-error rate $e_{ph}^{U}=n_{ph}^{U}/n_{2\mu }$.

## 4. Numerical Simulation

In this section, we simulate the final secret key rate with the parameters listed in Table 1.

It is reasonable to simulate the experimentally observed values $n_{2\mu }$, $n_{2\nu }$ and $n_{0}$ with their mean values. Let $Q_{corr|2\beta }$ be the probability of only one click from left (right) SPD when both Alice and Bob prepare coherent states with intensity β and a phase difference of 0 (π), and $Q_{err|2\beta }$ be the probability of only one click from left (right) SPD when both Alice and Bob prepare coherent states with intensity β and phase difference of π (0). Then, we have

(5) $$\begin{array}{c} Q_{corr|2\beta }=\left(1-\left(1-p_{d}\right)e^{-2\eta (1-e_{m})\beta }\right)e^{-2\eta e_{m}\beta }\left(1-p_{d}\right),\\ Q_{err|2\beta }=\left(1-\left(1-p_{d}\right)e^{-2\eta e_{m}\beta }\right)e^{-2\eta (1-e_{m})\beta }\left(1-p_{d}\right), \end{array}$$

where $\eta =10^{\frac{-0.2L}{20}}$ and *L* is the channel distance between Alice and Bob. Accordingly, in the simulation, we assume $n_{2\beta }=N_{tot}P_{\beta }^{2}2\left(Q_{corr|2\beta }+Q_{err|2\beta }\right)/M$ for β=μ,ν. Note that $n_{0}=N_{tot}P_{O}^{2}\left(Q_{corr|0}+Q_{err|0}\right)$, $n_{bit}=n_{2\mu }$ and $e_{bit}=Q_{err|2\mu }/\left(Q_{corr|2\mu }+Q_{err|2\mu }\right)$. With these values, setting M=8 and the failure probability of estimating phase error $\varepsilon =\left(6M+12\right)\varepsilon_{a}=4\times 10^{-20}$, one can obtain the upper-bound of phase-error rate $e_{ph}^{U}$ by the linear programming given by (A41) in Appendix A. Moreover, the amount of $H_{EC}$ is $H_{EC}=N_{bit}fh\left(e_{bit}\right)$, $\epsilon_{cor}=1\times 10^{-10}$, and $\epsilon_{PA}=1.6566\times 10^{-10}$, which leads to a secret key of length $l=n_{bit}\left(1-h\left(e_{ph}^{U}\right)\right)-H_{EC}-\log_{2}\frac{2}{\epsilon_{cor}}-\log_{2}\frac{1}{4\epsilon_{PA}^{2}}$ with $\epsilon_{sec}=\epsilon_{PA}+\sqrt{\varepsilon }$ and the total security parameter $\epsilon_{tol}=\epsilon_{cor}+\epsilon_{sec}=4.6566\times 10^{-10}$.

Finally, we numerically optimize the intensities and corresponding probabilities to maximize *l* in the cases of the total number of pulses is $N_{tot}=1\times 10^{17},1\times 10^{14},1\times 10^{13},1\times 10^{12}$. Note that because this numerical problem is very time-comsuming, these intensities and probabilities are not optimized at each distance. Additionally, we use some typical parameters instead. The simulate results ($l/N_{tot}$ v.s. L) are illustrated below.

**Figure 1:**
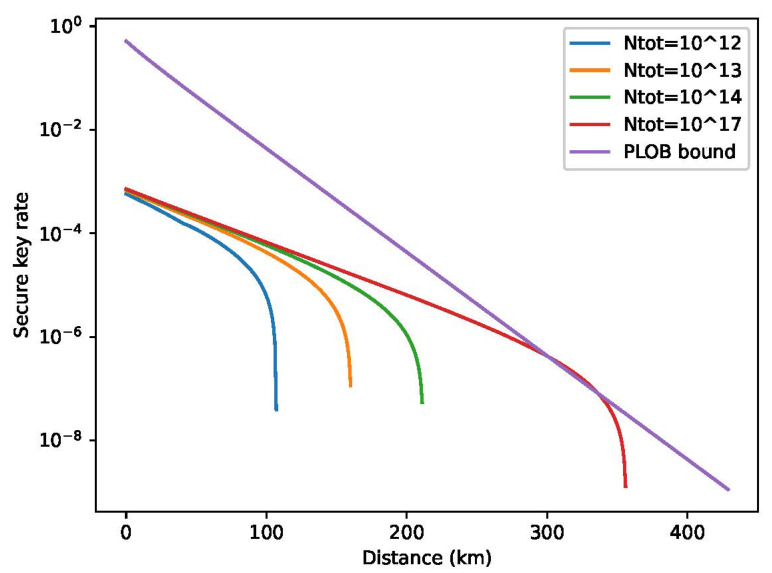
Secret key rate ($l/N_{tot}$) of fully discrete TF-QKD [43]. In this figure, the key rate corresponding to the total number of pulses $N_{tot}$ is $1\times 10^{12},1\times 10^{13},1\times 10^{14},1\times 10^{17}$, plotted above. Note that we set M=8 in the simulation.

As Figure 1 shows, we obtain considerable secret key rates when the total number of pulses is $10^{12}$, $10^{13}$, $10^{14}$ or $10^{17}$. Through numerical simulations, it is confirmed that TF-QKD with discrete-phase randomization has satisfactory performance. On the other hand, it is verified that finite-size effects become more notable here compared with the original protocol with continuous phase randomization; it seems that one has to prepare $10^{17}$ pulses to surpass the PLOB linear bound. This is because the statistical fluctuations in Lemma A1 are proportional to the square root of the total number of emitting pulse $N_{tot}$, which leads to alarge phase-error rate $e_{ph}$ when $n_{bit}$ is not sufficiently large.

## 5. Conclusions

In real setups of TF-QKD, continuous randomization is usually realized by actively adding a random signal to a phase modulator. On the other hand, random numbers are generated discretely in most schemes. Therefore, TF-QKD with discrete-phase randomization is more practical. It is necessary to analyze the security of TF-QKD with discrete-phase randomization. Based on conjugate measurement, the security proof of a QKD protocol is to estimate the phase-error rate. Then in case of discrete-phase randomization, a critical step is knowing how to bound the gap between yields of two distinct but very close quantum states in a non-asymptotic situation. To achieve this goal, Lemma A1 is developed to find the upper bound of this gap. With the help of Lemma 1, linear programming is proposed to calculate the phase-error rate, and the key length is then straightforward. Through numerical simulations, it is confirmed that TF-QKD with discrete-phase randomization has satisfactory performance. On the other hand, we also find that more pulses should be prepared to alleviate the finite-size effects than previous protocol.

Moreover, it is worth noting that Lemma A1 is quite useful in a variety of scenarios, not just in the security proof of TF-QKD. For instance, if one considers the BB84 with discrete-phase randomization [48], the Lemma A1 can be utilized to bound the yield of single photon state, so then it is not difficult to give a relevant security proof. To summarize, we give the first security proof for TF-QKD with finite discrete-phase randomization in non-asymptotic scenarios. Although the proof is tailored for TF-QKD, the framework of this proof, i.e. Lemma A1, can be adapted in other protocols.

## Figures and Tables

**Table 1:** List of parameters uesd in the numerical simulations. Here, $e_{m}$ is loss-independent misalignment error rate due to optical imperfect interference, $p_{d}$ is dark counting probability for each SPD, ξ is fiber loss constant, $\eta_{d}$ denotes detection efficiency of each SPD, *f* is error-correction inefficiency, and $\epsilon_{tol}$ denotes the total security coefficient.

$e_{m}$	$p_{d}$	ξ (dB/km)	$\eta_{d}$	*f*	$\epsilon_{\mathrm{tol}}$
0.03	$1\times 10^{-8}$	0.2	0.3	1.1	$4.6566\times 10^{-10}$

## Data Availability

Not applicable.

## Appendix A

### Appendix A.1. Formula for the Number of Phase-Error Events

In this section, we show how to obtain the relation between the hypothetical phase-error events and some experimental observations.

The main result obtained here is that each key bit is a successful click from a mixed state of $\tau_{j|2\mu },j=0,1,...,M-1$ prepared by Alice and Bob. More importantly, the number of phase-error events among these key bits corresponds to $\tau_{j|2\mu },j=0,2,...,M-2$. Therefore, if we denote the length of the raw key by $n_{bit}=n_{2\mu }$ and the number of successful clicks of $\tau_{j|2\mu }$ by $n_{j|2\mu }$, $n_{bit}=n_{2\mu }=\sum_{j=0}^{M-1}n_{j|2\mu }$, the number of phase-error events $n_{ph}=\sum_{j=0,j\in N_{0}}^{M-2}n_{j|2\mu }$ must hold, where $N_{0}$ is the set of even integers. Next, a proof is present to show how to obtain this result.

Following the symbols in [49], let us consider the evolution of the gigantic quantum state $|\Phi \rangle ={|\phi \rangle }^{⊗N_{tot}}={(|\psi \rangle }_{A_{s}A_{c}Aa}⊗{|\psi \rangle }_{B_{s}B_{c}Bb}{)}^{⊗N_{tot}}$ sent to Eve. After Step 2, where Eve performs her measurement on the subsystem ab, the initial quantum state is transformed to ${\hat{M}}_{eve}|\Phi \rangle$, where ${\hat{M}}_{eve}$ denotes the measurement operator of Eve. After measurement, Eve announces whether the measurement outcome is successful or not for each round. Hence, we reorder the quantum state as $|\Phi \rangle ={|\phi \rangle }^{⊗M}{|\phi \rangle }^{⊗\bar{M}}$, where $M(\bar{M})$ denotes the successful (unsuccessful) rounds. Then, in Step 3 of the virtual protocol, using measurement operators $\{{\hat{O}}_{s}={(|00\rangle }_{A_{s}B_{s}}\langle 00|+{|11\rangle }_{A_{s}B_{s}}\langle 11|+{|22\rangle }_{A_{s}B_{s}}\langle 22|)⊗\sum_{l=0}^{M-1}{(|l,l\rangle }_{A_{c}B_{c}}\langle l,l|+{|l,\left(l+M/2\right)modM\rangle }_{A_{c}B_{c}}\langle l,\left(l+M/2\right)modM|),{\hat{O}}_{d}=\hat{I}-{\hat{O}}_{s}\}$, Alice and Bob measure the subsystem $A_{s}A_{c}$ and $B_{s}B_{c}$ for those rounds that are announced successful in Step 2 and retain the trials in which $A_{s}A_{c}$ and $B_{s}B_{c}$ are collapsed into ${\hat{O}}_{s}$ as the final successful rounds. Hence, we reorder $|\Phi \rangle ={|\phi \rangle }^{⊗M_{s}}{|\phi \rangle }^{⊗M_{f}}{|\phi \rangle }^{⊗\bar{M}}$ where $M_{s}$($M_{f}$) denotes the successful(unsuccessful) rounds finally. Before they measure the subsystem AB to generate their sifted key in Step 3, the unnormalized quantum state is given by

(A1) $$\begin{array}{c} {\hat{O}}_{s}^{M_{s}}{\hat{O}}_{d}^{M_{f}}{\hat{I}}^{⊗\bar{M}}{\hat{M}}_{eve}|\Phi \rangle ={\hat{M}}_{eve}{\hat{O}}_{s}^{⊗M_{s}}{\hat{O}}_{d}^{⊗M_{f}}{\hat{I}}^{⊗\bar{M}}|\Phi \rangle ={\hat{M}}_{eve}({\hat{O}}_{s}|\phi \rangle {)}^{⊗M_{s}}({\hat{O}}_{f}|\phi \rangle {{)}^{⊗M_{f}}(|\phi \rangle )}^{⊗\bar{M}} \end{array}$$

Next, in Step 4, Alice and Bob measure the subsystem $A_{s},B_{s}$$A_{c},B_{c}$ and A,B for all rounds in $M_{s}$, one by one. We use $\alpha \in \{1,\cdots ,M_{s}\}$ to denote the different rounds in $M_{s}$ and $\xi_{\alpha }$ to denote the measurement outcome of the α-th subsystem. What is more, ${\hat{M}}_{\alpha }$ is used to denote the associated operator. Hence, the unnormalized state before the measurement of the α-th rounds in $M_{s}$ is

(A2) $$\begin{array}{c} |\Phi_{\alpha }\rangle ={\hat{M}}_{eve}({⊗}_{l=1}^{\alpha -1}{\hat{M}}_{l}|\phi \rangle )({\hat{O}}_{s}|\phi \rangle )({\hat{O}}_{s}|\phi \rangle {)}^{⊗M_{s}-\alpha }({\hat{O}}_{f}|\phi \rangle {{)}^{⊗M_{f}}(|\phi \rangle )}^{⊗\bar{M}}. \end{array}$$

because we are only interested in the reduced state of the α-th round in $M_{s}$, we trace out the other rounds which we denote by $\bar{\alpha }$ and obtain

(A3) $$\begin{array}{c} {\hat{\sigma }}_{\alpha }=Tr_{\bar{\alpha }}\left[|\Phi_{\alpha }\rangle \langle |\Phi_{\alpha }|\right]=\sum_{\vec{\bar{\alpha }}}\langle \vec{\bar{\alpha }}|\Phi_{\alpha }\rangle \langle \Phi_{\alpha }|\vec{\bar{\alpha }}\rangle =\sum_{\vec{\bar{\alpha }}}{\hat{M}}_{\vec{\bar{\alpha }}}{\hat{O}}_{s}\left|\phi \rangle \langle \phi \right|{\hat{O}}_{s}^{†}{\hat{M}}_{\vec{\bar{\alpha }}}^{†} \end{array}$$

where

(A4) $$\begin{array}{c} {\hat{M}}_{\vec{\bar{\alpha }}}=\langle \vec{\bar{\alpha }}|{\hat{M}}_{eve}|\left({⊗}_{l=1}^{\alpha -1}{\hat{M}}_{l}({\hat{O}}_{s}|\phi \rangle \right)({\hat{O}}_{s}|\phi \rangle {)}^{⊗M_{s}-\alpha }({\hat{O}}_{f}|\phi \rangle {{)}^{⊗M_{f}}(|\phi \rangle )}^{⊗\bar{M}}. \end{array}$$

and the quantum states $\left\{|\vec{\bar{\alpha }}\rangle \right\}$ represent the basis for the subsystems $A_{s},B_{s},A_{c},B_{c},A,B,a,b$ of all rounds in the protocol except the α-th round in $M_{s}$.

Next, to derive the number of phase error, we expand the quantum state ${\hat{O}}_{s}|\phi \rangle$ as

(A5) $$\begin{array}{c} {\hat{O}}_{s}|\phi \rangle =p_{\mu }{|00\rangle }_{A_{s}B_{s}}{|\phi \rangle }_{\mu }+p_{O}{|11\rangle }_{A_{s}B_{s}}{|\phi \rangle }_{0}+p_{\nu }{|22\rangle }_{A_{s}B_{s}}{|\phi \rangle }_{\nu } \end{array}$$

where

$$\begin{array}{cc} & {|\phi \rangle }_{\mu }\\ = & \sum_{l=0}^{M-1}\frac{1}{M}{[{(|ll\rangle }_{A_{c}B_{c}}\frac{1}{2}{(|0\rangle }_{A}|e^{i\frac{2\pi }{M}l}\sqrt{\mu }\rangle }_{a}+{|1\rangle }_{A}|-e^{i\frac{2\pi }{M}l}\sqrt{\mu }\rangle_{a}{)(|0\rangle }_{B}|e^{i\frac{2\pi }{M}l}\sqrt{\mu }\rangle_{b}+{|1\rangle }_{B}|-e^{i\frac{2\pi }{M}l}\sqrt{\mu }\rangle_{b}))\\ & +{(|l,\left(l+\frac{M}{2}\right)modM\rangle }_{A_{c}B_{c}}\frac{1}{2}{(|0\rangle }_{A}|e^{i\frac{2\pi }{M}l}\sqrt{\mu }\rangle_{a}+{|1\rangle }_{A}|-e^{i\frac{2\pi }{M}l}\sqrt{\mu }\rangle_{a}){(|0\rangle }_{B}|-e^{i\frac{2\pi }{M}l}\sqrt{\mu }\rangle_{b}+{|1\rangle }_{B}|e^{i\frac{2\pi }{M}l}\sqrt{\mu }\rangle_{b})))], \end{array}$$

(A6) $$\begin{array}{c} {|\phi \rangle }_{0}={|00\rangle }_{A_{c}B_{c}}{|00\rangle }_{AB}{|00\rangle }_{ab} \end{array}$$

and

(A7) $$\begin{array}{cc} & {|\phi \rangle }_{\nu }\\ = & \sum_{l=0}^{M-1}\frac{1}{M}{[{(|ll\rangle }_{A_{c}B_{c}}{(|00\rangle }_{AB}|e^{i\frac{2\pi }{M}l}\sqrt{\nu }\rangle }_{a}|e^{i\frac{2\pi }{M}l}\sqrt{\nu }\rangle_{b})+{(|l,\left(l+\frac{M}{2}\right)modM\rangle }_{A_{c}B_{c}}{|00\rangle }_{AB}{(|e^{i\frac{2\pi }{M}l}\sqrt{\nu }\rangle }_{a}|-e^{i\frac{2\pi }{M}l}\sqrt{\nu }\rangle_{b}))]. \end{array}$$

To summarize, each key bit can be viewed as an event in which Eve announces a successful click conditioned by Alice and prepares ${|\phi \rangle }_{\mu }$ and measures AB with *Z*-basis. Since the measurement on AB made by Alice and Bob can be delayed after Eve’s announcement of a successful click, the phase error can be estimated by Alice and Bob measuring AB with *X*-basis rather than *Z*-basis. To obtain the phase error of this part, we rewrite ${|\phi \rangle }_{\mu }$ under *X*-bases of AB as

(A8) $$\begin{array}{c} {|\phi \rangle }_{\mu }=\sum_{l=0}^{M-1}\frac{1}{M}{(|ll\rangle }_{A_{c}B_{c}}{|\psi \rangle }_{\mu ,AaBb}^{l}+|l,\left(l+\frac{M}{2}\right){modM\rangle }_{A_{c}B_{c}}|\psi^{\prime }\rangle_{\mu ,AaBb}^{l}) \end{array}$$

where

(A9) $$\begin{array}{cc} \; & {|\psi \rangle }_{\mu ,AaBb}^{l}\\ = & \frac{1}{2}{(|00\rangle }_{AB}|e^{i\theta_{l}}\sqrt{\mu }\rangle_{a}|e^{i\theta_{l}}\sqrt{\mu }\rangle_{b}+{|01\rangle }_{AB}|e^{i\theta_{l}}\sqrt{\mu }\rangle_{a}|-e^{i\theta_{l}}\sqrt{\mu }\rangle_{b})\\ + & \frac{1}{2}{(|10\rangle }_{AB}|-e^{i\theta_{l}}\sqrt{\mu }\rangle_{a}|e^{i\theta_{l}}\sqrt{\mu }\rangle_{b}+{|11\rangle }_{AB}|-e^{i\theta_{l}}\sqrt{\mu }\rangle_{a}|-e^{i\theta_{l}}\sqrt{\mu }\rangle_{b})\\ = & \frac{1}{4}{|++\rangle }_{AB}{(|e^{i\theta_{l}}\sqrt{\mu }\rangle }_{a}|e^{i\theta_{l}}\sqrt{\mu }\rangle_{b}+|e^{i\theta_{l}}\sqrt{\mu }\rangle_{a}|-e^{i\theta_{l}}\sqrt{\mu }\rangle_{b}+|-e^{i\theta_{l}}\sqrt{\mu }\rangle_{a}|e^{i\theta_{l}}\sqrt{\mu }\rangle_{b}+|-e^{i\theta_{l}}\sqrt{\mu }\rangle_{a}|-e^{i\theta_{l}}\sqrt{\mu }\rangle_{b})\\ + & \frac{1}{4}{|--\rangle }_{AB}{(|e^{i\theta_{l}}\sqrt{\mu }\rangle }_{a}|e^{i\theta_{l}}\sqrt{\mu }\rangle_{b}-|e^{i\theta_{l}}\sqrt{\mu }\rangle_{a}|-e^{i\theta_{l}}\sqrt{\mu }\rangle_{b}-|-e^{i\theta_{l}}\sqrt{\mu }\rangle_{a}|e^{i\theta_{l}}\sqrt{\mu }\rangle_{b}+|-e^{i\theta_{l}}\sqrt{\mu }\rangle_{a}|-e^{i\theta_{l}}\sqrt{\mu }\rangle_{b})\\ + & \frac{1}{4}{|+-\rangle }_{AB}\left(\cdots \right)+\frac{1}{4}{|-+\rangle }_{AB}\left(\cdots \right)), \end{array}$$

(A10) $$\begin{array}{cc} \; & |\psi^{\prime }\rangle_{\mu ,AaBb}^{l}\\ = & \frac{1}{2}{(|00\rangle }_{AB}|e^{i\theta_{l}}\sqrt{\mu }\rangle_{a}|-e^{i\theta_{l}}\sqrt{\mu }\rangle_{b}+{|01\rangle }_{AB}|e^{i\theta_{l}}\sqrt{\mu }\rangle_{a}{|e^{i\theta_{l}}\sqrt{\mu }\rangle }_{b}\\ + & \frac{1}{2}{|10\rangle }_{AB}|-e^{i\theta_{l}}\sqrt{\mu }\rangle_{a}|-e^{i\theta_{l}}\sqrt{\mu }\rangle_{b}+{|11\rangle }_{AB}|-e^{i\theta_{l}}\sqrt{\mu }\rangle_{a}|e^{i\theta_{l}}\sqrt{\mu }\rangle_{b})\\ = & \frac{1}{4}{|++\rangle }_{AB}{(|e^{i\theta_{l}}\sqrt{\mu }\rangle }_{a}|-e^{i\theta_{l}}\sqrt{\mu }\rangle_{b}+|e^{i\theta_{l}}\sqrt{\mu }\rangle_{a}|e^{i\theta_{l}}\sqrt{\mu }\rangle_{b}+|-e^{i\theta_{l}}\sqrt{\mu }\rangle_{a}|-e^{i\theta_{l}}\sqrt{\mu }\rangle_{b}+|-e^{i\theta_{l}}\sqrt{\mu }\rangle_{a}|e^{i\theta_{l}}\sqrt{\mu }\rangle_{b})\\ + & \frac{1}{4}{|--\rangle }_{AB}{(|e^{i\theta_{l}}\sqrt{\mu }\rangle }_{a}|-e^{i\theta_{l}}\sqrt{\mu }\rangle_{b}-|e^{i\theta_{l}}\sqrt{\mu }\rangle_{a}|e^{i\theta_{l}}\sqrt{\mu }\rangle_{b}-|-e^{i\theta_{l}}\sqrt{\mu }\rangle_{a}|-e^{i\theta_{l}}\sqrt{\mu }\rangle_{b}+|-e^{i\theta_{l}}\sqrt{\mu }\rangle_{a}|e^{i\theta_{l}}\sqrt{\mu }\rangle_{b})\\ + & \frac{1}{4}{|+-\rangle }_{AB}\left(\cdots \right)+\frac{1}{4}{|-+\rangle }_{AB}\left(\cdots \right)), \end{array}$$

and $\theta_{l}=\frac{l}{M}2\pi$. For the purpose of clarification, we define some quantum states below:

(A11) $$\begin{array}{c} |e^{i\theta }\sqrt{2\mu }\rangle_{ab}=\sum_{j=0}^{\infty }e^{ij\theta }\sqrt{P_{j|2\mu }}{|j\rangle }_{ab} \end{array}$$

and

(A12) $$\begin{array}{c} |e^{i\theta }\sqrt{2\mu }\rangle_{ab,even}=\frac{|e^{i\theta }\sqrt{\mu }\rangle_{a}|e^{i\theta }\sqrt{\mu }\rangle_{b}+|-e^{i\theta }\sqrt{\mu }\rangle_{a}{|-e^{i\theta }\sqrt{\mu }\rangle }_{b}}{2}=\sum_{j\in N_{0}}e^{ij\theta }\sqrt{P_{j|2\mu }}{|j\rangle }_{ab}, \end{array}$$

where ${|j\rangle }_{ab}=\sum_{i=0}^{j}\sqrt{\frac{j!}{2^{j}i!\left(j-i\right)!}}{|i\rangle }_{a}{|j-i\rangle }_{b}$ and $N_{0}$ is the set of even numbers. Indeed, |j〉 is a quantum state satisfying that the total photon-number of *a* and *b* is *j*. Moreover, another similar quantum state is defined below:

(A13) $$\begin{array}{c} |e^{i\theta }\sqrt{2\mu }\rangle_{ab}^{\prime }=\sum_{j=0}^{\infty }e^{ij\theta }\sqrt{P_{j|2\mu }}{|j\rangle }_{ab}^{\prime } \end{array}$$

and

(A14) $$\begin{array}{c} |e^{i\theta }\sqrt{2\mu }\rangle_{ab}^{\prime }=\frac{|e^{i\theta }\sqrt{\mu }\rangle_{a}|-e^{i\theta }\sqrt{\mu }\rangle_{b}+|-e^{i\theta }\sqrt{\mu }\rangle_{a}{|e^{i\theta }\sqrt{\mu }\rangle }_{b}}{2}=\sum_{j\in N_{0}}e^{ij\theta }\sqrt{P_{j|2\mu }}{|j\rangle }_{ab}^{\prime } \end{array}$$

where ${|j\rangle }_{ab,even}^{\prime }=\sum_{i=0}^{j}{\left(-1\right)}^{i}\sqrt{\frac{j!}{2^{j}i!\left(j-i\right)!}}{|i\rangle }_{a}{|j-i\rangle }_{b}$. With these definitions, we can write the quantum states ${|\psi \rangle }_{\mu ,AaBb}^{l}$ and $|\psi^{\prime }\rangle_{\mu ,AaBb}^{l}$ in a more simplified way, namely

(A15) $$\begin{array}{cc} {|\psi \rangle }_{\mu ,AaBb}^{l}= & \frac{1}{2}{(|++\rangle }_{AB}{(|e^{i\theta_{l}}\sqrt{2\mu }\rangle }_{ab,even}+|e^{i\theta_{l}}\sqrt{2\mu }\rangle_{ab,even}^{\prime }{)+|--\rangle }_{AB}{(|e^{i\theta_{l}}\sqrt{2\mu }\rangle }_{ab,even}-|e^{i\theta_{l}}\sqrt{2\mu }\rangle_{ab,even}^{\prime })\\ \; & {+|+-\rangle }_{AB}{\left(\cdots \right)+|-+\rangle }_{AB}\left(\cdots \right)) \end{array}$$

and

(A16) $$\begin{array}{cc} |\psi^{\prime }\rangle_{\mu ,AaBb}^{l}= & \frac{1}{2}{(|++\rangle }_{AB}{(|e^{i\theta_{l}}\sqrt{2\mu }\rangle }_{ab,even}+|e^{i\theta_{l}}\sqrt{2\mu }\rangle_{ab,even}^{\prime }{)-|--\rangle }_{AB}{(|e^{i\theta_{l}}\sqrt{2\mu }\rangle }_{ab,even}-|e^{i\theta_{l}}\sqrt{2\mu }\rangle_{ab,even}^{\prime })\\ \; & {+|+-\rangle }_{AB}{\left(\cdots \right)+|-+\rangle }_{AB}\left(\cdots \right)) \end{array}$$

obviously, the measurement outcome of ${|++\rangle }_{AB}$ and ${|--\rangle }_{AB}$ can be defined as phase-error event. Recall the whole density matrix ${|\phi \rangle }_{\mu }\langle \phi |$ given in Equation (A8); we can obtain the ab part corresponding to the phase-error event,

(A17) $$\begin{array}{cc} {\hat{\rho }}_{\mu ,ph}= & {\langle ++|}_{AB}tr_{A_{c}B_{c}}{(|\phi \rangle }_{\mu }{\langle \phi |)|++\rangle }_{AB}{+\langle --|}_{AB}tr_{A_{c}B_{c}}{(|\phi \rangle }_{\mu }\langle \phi |){|--\rangle }_{AB}\\ = & \sum_{l=0}^{M-1}{\langle ll|}_{A_{c}B_{c}}{\left(\langle ++\right|}_{AB}+\langle --{|}_{AB}){|\phi \rangle }_{\mu }\langle \phi |{(|++\rangle }_{AB}{+|--\rangle }_{AB}{)|ll\rangle }_{A_{c}B_{c}}\\ + & {\langle l,\left(l+M/2\right)modM|}_{A_{c}B_{c}}{\left(\langle ++\right|}_{AB}+\langle --{|}_{AB}){|\phi \rangle }_{\mu }\langle \phi |{(|++\rangle }_{AB}{+|--\rangle }_{AB})|l,\left(l+M/2\right){modM\rangle }_{A_{c}B_{c}}\\ = & \sum_{l=0}^{M-1}\frac{1}{M^{2}}{(|e^{i\theta_{l}}\sqrt{2\mu }\rangle }_{ab}\langle e^{i\theta_{l}}\sqrt{2\mu }\left|+\right|e^{i\theta_{l}}\sqrt{2\mu }\rangle_{ab}^{\prime }\langle e^{i\theta_{l}}\sqrt{2\mu }|) \end{array}$$

according to Equations (2.5)–(2.7) of Ref [48], ${\hat{\rho }}_{\mu ,ph}$ can be rewritten as

(A18) $$\begin{array}{cc} {\hat{\rho }}_{\mu ,ph}= & \frac{2}{M}\sum_{j=0,j\in N_{0}}^{M-2}{\tilde{P}}_{j|2\mu }{(\frac{1}{2}|{\tilde{j}}_{2\mu }\rangle }_{ab}\langle {\tilde{j}}_{2\mu }|+\frac{1}{2}{|{\tilde{j}}_{2\mu }\rangle }_{ab}^{\prime }\langle {\tilde{j}}_{2\mu }|)\\ = & \frac{2}{M}\sum_{j=0,j\in N_{0}}^{M-2}{\tilde{P}}_{j|2\mu }\tau_{j|2\mu }, \end{array}$$

where ${\tilde{P}}_{j|2\mu }=\sum_{n=0}^{\infty }P_{j+Mn|2\mu }$, $P_{j|2\mu }$ is the the probability of finding *j* photons in a Poisson source with mean photon-number 2μ, $|{\tilde{j}}_{2\mu }\rangle =\sum_{n=0}^{\infty }\frac{\sqrt{P_{j+Mn|2\mu }}}{\sqrt{{\tilde{P}}_{j|2\mu }}}{|j+Mn\rangle }_{ab}$ and $|{\tilde{j}}_{2\mu }\rangle^{\prime }=\sum_{n=0}^{\infty }\frac{\sqrt{P_{j+Mn|2\mu }}}{\sqrt{{\tilde{P}}_{j|2\mu }}}{|j+Mn\rangle }_{ab}^{\prime }$, and $\tau_{j|2\mu }=\frac{1}{2}|{\tilde{j}}_{2\mu }\rangle_{ab}\langle {\tilde{j}}_{2\mu }|+\frac{1}{2}|{\tilde{j}}_{2\mu }\rangle_{ab}^{\prime }\langle {\tilde{j}}_{2\mu }|$.

For ease of understanding, we can easily interpret the formula of ${\hat{\rho }}_{\mu ,ph}$. It is easy to see that ${\hat{\rho }}_{\mu ,ph}$ is a mixture of $\tau_{j|2\mu }$, which consists of photon-number state ${|j+Mn\rangle }_{ab},n=0,1,2,...$, and the probability of finding j+Mn photons is proportional to $P_{j+Mn|2\mu }$. Let $\tau_{even|2\mu }$ be the normalized ${\hat{\rho }}_{\mu ,ph}$; then, a phase-error event for a key bit is equivalent to a successful click announced by Eve on the condition that a mixture $\tau_{even|2\mu }=\sum_{j=0,j\in N_{0}}^{M-2}{\tilde{P}}_{j|2\mu }\tau_{j|2\mu }/$$P_{even|2\mu }$ prepared by Alice and Bob, and the probability of preparing such a mixture is obviously $P_{\mu }^{2}P_{even|2\mu }2/M$.

To find a way to estimate the number of phase errors, we can give the density matrices Alice and Bob prepared in code mode and decoy mode. If we trace out $ABA_{c}B_{c}$ of the quantum state ${|\phi \rangle }_{\mu }\langle \phi |$, i.e., regardless of whether the measurement outcome on AB is phase error or not, we have

(A19) $$\begin{array}{cc} {\hat{\rho }}_{\mu }= & Tr_{ABA_{c}B_{c}}{(|\phi \rangle }_{\mu }\langle \phi |)\\ = & \frac{2}{M}\sum_{j=0}^{M-1}{\tilde{P}}_{j|2\mu }(\frac{1}{2}|{\tilde{j}}_{2\mu }\rangle \langle {\tilde{j}}_{2\mu }|+\frac{1}{2}{|{\tilde{j}}_{2\mu }\rangle }^{\prime }\langle {\tilde{j}}_{2\mu }|)\\ = & \frac{2}{M}\sum_{j=0}^{M-1}{\tilde{P}}_{j|2\mu }\tau_{j|2\mu }. \end{array}$$

we define $\tau_{2\mu }=\sum_{j=0}^{M-1}{\tilde{P}}_{j|2\mu }\tau_{j|2\mu }$ as the normalized ${\hat{\rho }}_{\mu }$. The generation of a key bit is equivalent to a successful click announced by Eve conditioned on the premise that a mixture $\tau_{2\mu }=\sum_{j=0}^{M-1}{\tilde{P}}_{j|2\mu }\tau_{j|2\mu }$ is prepared by Alice and Bob, and the probability of preparing such a mixture is obviously $P_{\mu }^{2}2/M$.

Now we are approaching a main result of above derivations. In the $N_{tot}$ rounds, Alice and Bob prepare $\tau_{2\mu }$ with the probability $P_{\mu }^{2}2/M$; the number of its successful rounds is denoted by $n_{2\mu }$. Of course, the key bits are generated in these rounds, thus $n_{2\mu }=n_{bit}$. Since $\tau_{2\mu }$ is a mixture of $\tau_{j|2\mu },j=0,1...,,M-1$, the $n_{2\mu }$ successful clicks are the sum of clicks by $\tau_{j|2\mu },j=0,1,...,M-1$. Then, $n_{2\mu }=\sum_{j=0}^{M-1}n_{j|2\mu }$ holds evidently, in which $n_{j|2\mu }$ is the number of successful clicks by $\tau_{j|2\mu }$. Recall that the phase errors for these events are from clicks by $\tau_{even|2\mu }$; one can assert that the number of phase-error events $n_{ph}=\sum_{j=0,j\in N_{0}}^{M-2}n_{j|2\mu }$ must hold. This is a main result we have so far.

### Appendix A.2. The Upper Bound of the Number of Phase-Error Events

We have proved that $n_{ph}=\sum_{j=0,j\in N_{0}}^{M-2}n_{j|2\mu }$ with the constraint $n_{2\mu }=\sum_{j=0}^{M-1}n_{j|2\mu }$. Obviously, this is not sufficient for estimating $n_{ph}$ tightly. Here, we resort to decoy states to obtain more constraints to bound $n_{ph}$.

Similarly with the analysis of clicks by ${\hat{\rho }}_{\mu }=Tr_{ABA_{c}B_{c}}{(|\phi \rangle }_{\mu }\langle \phi |)$, a successful click from decoy mode with intensity ν means that Alice and Bob prepare

(A20) $$\begin{array}{cc} {\hat{\rho }}_{\nu }= & Tr_{ABA_{c}B_{c}}{(|\phi \rangle }_{\nu }\langle \phi |)\\ = & \frac{2}{M}\sum_{j=0}^{M-1}{\tilde{P}}_{j|2\nu }(\frac{1}{2}|{\tilde{j}}_{2\nu }\rangle \langle {\tilde{j}}_{2\nu }|+\frac{1}{2}{|{\tilde{j}}_{2\nu }\rangle }^{\prime }\langle {\tilde{j}}_{2\nu }|)\\ = & \frac{2}{M}\sum_{j=0}^{M-1}{\tilde{P}}_{j|2\nu }\tau_{j|2\nu }. \end{array}$$

accordingly, we also have $n_{2\nu }=\sum_{j=0}^{M-1}n_{j|2\nu }$. Here, $n_{2\nu }$ and $n_{j|2\nu }$ are defined analogously to $n_{2\mu }$ and $n_{j|2\mu }$, respectively. Intuitively, $n_{j|2\mu }$ and $n_{j|2\mu }$ are the numbers of successful clicks for $\tau_{j|2\mu }$ and $\tau_{j|2\nu }$ respectively. Typically, μ<ν<<1 is satisfied; then both $\tau_{j|2\mu }$ and $\tau_{j|2\nu }$ are very close to the photon-number state ${|j\rangle }_{ab}$. This implies that the gap between $n_{j|2\mu }$ and $n_{j|2\nu }$ can be bounded, and then we may estimate $n_{ph}=\sum_{j=0,j\in N_{0}}^{M-2}n_{j|2\mu }$. Indeed, with the result in appendix B of ref [48], the gap between $n_{j|2\mu }$ and $n_{j|2\nu }$ in the asymptotic case can be obtained. Here, we develop Lemma A1 to bound this gap in finite-key situations.

**Lemma** **A1.**
*If Alice prepares $N_{tot}$ pairs of particles A and B with the quantum state ${(\sum_{i}\sqrt{P_{i}}|i\rangle }_{A}|\phi_{i}\rangle_{B})$${}^{⊗N_{tot}}$ where $\langle i|j\rangle =\delta_{ij},\left|\langle \phi_{i}|\phi_{j}\rangle \right|=F_{ij}$ and she sends the B part in each pair to Eve. For every round, Eve announces if the measurement is successful or unsuccessful, which is denoted by M=1 or M=0, respectively. Then, Alice measures the subsystem A with projectors {|i〉〈i|,i=0,1,2,...} to which quantum state she sent for the pairs that Eve announced M=1. Let $n_{i}$ denote the number of yields for the quantum state |i〉. If $P_{i}>P_{j}$, we have that the constraints between $n_{i}$ and $n_{j}$, say,*

(A21)
$$\begin{array}{c} \left|\frac{P_{j}}{P_{i}}n_{i}-n_{j}\right|\leq N_{1}\sqrt{1-F_{ij}^{2}}+2\delta \left(N_{1},\frac{1+\sqrt{1-F_{ij}^{2}}}{2},\varepsilon_{0}^{2}\right)-2\delta \left(N_{2}-n_{i}-n_{j},\frac{1}{2},\varepsilon_{0}\right)+\delta \left(n_{i},\frac{P_{j}}{P_{i}},\varepsilon_{2}\right) \end{array}$$

*holds with a failure probability $2\varepsilon_{0}+2\varepsilon_{1}+2\varepsilon_{2}$, where*

(A22)
$$\begin{array}{cc} \delta (x,y,z)= & \sqrt{3xy\ln(\frac{1}{z})}\\ N_{1}= & 2P_{j}N_{tot}+\delta \left(N_{tot},2P_{j},\varepsilon_{1}\right)\\ N_{2}= & 2P_{j}N_{tot}-\delta \left(N_{tot},2P_{j},\varepsilon_{1}\right) \end{array}$$

**Proof.** Since we are only interested in the statistics of $n_{i}$ and $n_{j}$, it is not restrictive to rewrite the quantum state ${(\sum_{i}\sqrt{P_{i}}|i\rangle }_{A}|\phi_{i}{\rangle_{B})}^{⊗N_{tot}}$ as

(A23) $$\begin{array}{c} (\sum_{i}\sqrt{P_{i}}|i\rangle_{A}|\phi_{i}\rangle_{B}{)}^{⊗N_{tot}}={\{\sqrt{P_{i}-P_{j}}|i^{\prime }\rangle }_{A}|\phi_{i}\rangle_{B}+\sqrt{2P_{j}}\frac{1}{\sqrt{2}}{(|i^{{}^{\prime \prime }}\rangle }_{A}|\phi_{i}\rangle_{B}+{|j\rangle }_{A}|\phi_{j}\rangle_{B}{)+\ldots \}}^{⊗N_{tot}}. \end{array}$$

Here, we virtually define $\sqrt{P_{i}}{|i\rangle }_{A}=\sqrt{P_{i}-P_{j}}|i^{\prime }\rangle_{A}+\sqrt{P_{j}}{|i^{\prime \prime }\rangle }_{A}$ and ${\langle i^{\prime }|i^{\prime \prime }\rangle }_{A}=0$, which do not change the density matrix of *B*; thus, have no impact on Eve’s operation and statistics of $n_{i}$ and $n_{j}$. Let $n_{i^{\prime }}$($n_{i^{\prime \prime }}$) denote the number of detection for the quantum state $|i^{\prime }\rangle_{A}$($|i^{\prime \prime }\rangle_{A}$) when Eve announces a successful measurement. Apparently, we know that $n_{i}=n_{i^{\prime }}+n_{i^{\prime \prime }}$. Let us focus on the state ${\{\sqrt{2P_{j}}\frac{1}{\sqrt{2}}{(|i^{{}^{\prime \prime }}\rangle }_{A}|\phi_{i}\rangle }_{B}+{|j\rangle }_{A}|\phi_{j}\rangle_{B}{)\}}^{⊗N_{tot}}$, by which the relation between $n_{i^{\prime \prime }}$ and $n_{j}$ can be analyzed. The essential idea is reinterpreting the detection of *B* to a game of Eve guessing which states $|\phi_{i}\rangle_{B}$ or $|\phi_{j}\rangle_{B}$ Alice prepared for all of the $N_{tot}$ trials. Specifically, we consider a virtual experiment illustrated below. Alice prepares the quantum state ${\{\sqrt{2P_{j}}\frac{1}{\sqrt{2}}{(|i^{{}^{\prime \prime }}\rangle }_{A}|\phi_{i}\rangle }_{B}+{|j\rangle }_{A}|\phi_{j}\rangle_{B}{)\}}^{⊗N_{tot}}$; then, she sends the *B* part to Eve. Eve measures each *B* she received. If she obtains a successful measurement, she will announce M=1. Otherwise, she will announce M=0. Up to now, there is no difference from the previous protocol. A critical step is that for any trial that Eve announces M=0, Alice flips corresponding *M* with probability $\frac{1}{2}$. Finally, Alice measures all partials *A* locally. It is obvious that if Alice prepares two quantum states $|\phi_{i}\rangle$ and $|\phi_{j}\rangle$ at random, the maximal probability of Eve guessing correctly which state is prepared is $\frac{1+\sqrt{1-F_{ij}^{2}}}{2}$ where $F_{ij}=\left|\langle \phi_{i}|\phi_{j}\rangle \right|$. With the flip operation, we can apply this maximal probability to our analysis below. In this case, we reinterpret that M=1 (M=0) means that Eve guessed the quantum state Alice prepared is $|\phi_{i}\rangle$ ($|\phi_{j}\rangle$), which justifies the lossless assumption. Note that this does not compromise security because as an adversary, Eve can guess in this way. In other words, we can now treat this virtual experiment as a game where Eve tries to guess whether Alice is preparing $|\phi_{i}\rangle$ or $|\phi_{j}\rangle$. For each of all the trials in such a game, it is well known that Eve’s maximal probability of guessing correctly is $\frac{1+\sqrt{1-F_{ij}^{2}}}{2}$. Now, we are ready to find the relation between $n_{i^{\prime \prime }}$ and $n_{j}$ by calculating how many trials in which Eve’s guessing is correct. First, $n_{i^{\prime \prime }}$ means that announcing M=1 at first and Alice also preparing $|\phi_{i}\rangle_{B}$, which of course leads to guessing correctly. Then, let $N_{i^{\prime \prime }}$($N_{j}$) denote Alice preparing the state $|\phi_{i}\rangle$($|\phi_{j}\rangle$), which implies that $N_{i^{\prime \prime }}+N_{j}\approx N_{tot}2P_{j}$. As a result, there are $\left(N_{i^{\prime \prime }}+N_{j}-n_{i^{\prime \prime }}-n_{j}\right)$ trials in which Eve announces M=0 at first and then a random flipping operation on *M* follows; for every such trial, the probability of guessing correctly is obviously 1/2. Further considering the potential statistical fluctuations made by the random flipping, with a failure probability of $\varepsilon_{1}^{\prime }$, the number of Eve guessing correctly in the $N_{i^{\prime \prime }}+N_{j}$ trials is no larger than

(A24) $$\begin{array}{c} n_{i^{\prime \prime }}+\frac{1}{2}\left(N_{i^{\prime \prime }}-n_{i^{\prime \prime }}\right)+\frac{1}{2}\left(N_{j}-n_{j}\right)+\bar{\delta_{1}}, \end{array}$$

where $\bar{\delta_{1}}=\delta \left(N_{i^{\prime \prime }}+N_{j}-n_{i^{\prime \prime }}-n_{j},\frac{1}{2},\varepsilon_{1}^{\prime }\right)$ is the upper bound of the statistical fluctuation made by the random flipping of the $\left(N_{i^{\prime \prime }}+N_{j}-n_{i^{\prime \prime }}-n_{j}\right)$ trials. We let $\varepsilon_{1}^{\prime }$ be the probability of the amount of successful detection of quantum state $|i^{\prime \prime }\rangle$ reach $n_{i^{\prime \prime }}$. Hence the probability that we get the quantity denoted by Equarion (A24) is $\varepsilon_{1}^{{}^{\prime }2}$. On the other hand, in the $N_{i^{\prime \prime }}+N_{j}$ trials of guessing $|\phi_{i}\rangle$ and $|\phi_{j}\rangle$ prepared by Alice at random, the probability of Eve guessing correctly is no larger than $\frac{1+\sqrt{1-F_{ij}^{2}}}{2}$ [50], because the fidelity of $|\phi_{i}\rangle$ and $|\phi_{j}\rangle$ is $F_{ij}$, Hence, one can assert that when $\varepsilon_{1}^{{}^{\prime }2}\leq \varepsilon_{0}^{2}$, In this case, we let $\varepsilon_{1}^{\prime }=\varepsilon_{0}$. Hence, one can assert that with a failure probability $\varepsilon_{0}$,

(A25) $$\begin{array}{c} \frac{n_{i^{\prime \prime }}-n_{j}}{2}+\frac{N_{i^{\prime \prime }}+N_{j}}{2}+\bar{\delta_{1}}\leq \left(N_{i^{\prime \prime }}+N_{j}\right)\frac{1+\sqrt{1-F_{ij}^{2}}}{2}+\bar{\delta_{2}} \end{array}$$

holds, where $\bar{\delta_{2}}=\delta \left(N_{i^{\prime \prime }}+N_{j},\frac{1+\sqrt{1-F_{ij}^{2}}}{2},\varepsilon_{0}^{2}\right)$ is the upper bound of the statistical fluctuation when Eve’s guessing probability for each trial achieves the upper-bound $\frac{1+\sqrt{1-F_{ij}^{2}}}{2}$. Note that because in such a guessing game, conditioned on other trials, the probability of guessing correctly for any fixed trial cannot be larger than $\frac{1+\sqrt{1-F_{ij}^{2}}}{2}$, this lemma is applied to any attack. Furthermore, the probability of guessing correctly reaches its maximum, which equals a constant; then, the assumpution of iid holds and the Bernoulli distribution is applied to this case. However, since it is hard to calculate the statistical fluctuation, we use the Chernoff bound $\delta_{2}$ to approximate it [51]. According to Equation (A25), we can obtain an upper bound of $n_{i^{\prime \prime }}-n_{j}$ with a failure probability $\varepsilon_{0}$, say

(A26) $$\begin{array}{c} n_{i^{\prime \prime }}-n_{j}\leq \left(N_{i^{\prime \prime }}+N_{j}\right)\sqrt{1-F_{ij}^{2}}+2\delta \left(N_{i^{\prime \prime }}+N_{j},\frac{1+\sqrt{1-F_{ij}^{2}}}{2},\varepsilon_{0}^{2}\right)-2\delta \left(N_{i^{\prime \prime }}+N_{j}-n_{i^{\prime \prime }}-n_{j},\frac{1}{2},\varepsilon_{0}\right). \end{array}$$

similarly, if we redefine the guessing correctly as M=1 corresponding to $|\phi_{j}\rangle$ and M=0 corresponding to $|\phi_{i}\rangle$, we have that

(A27) $$\begin{array}{c} n_{j}-n_{i}^{\prime \prime }\leq \left(N_{i^{\prime \prime }}+N_{j}\right)\sqrt{1-F_{ij}^{2}}+2\delta \left(N_{i^{\prime \prime }}+N_{j},\frac{1+\sqrt{1-F_{ij}^{2}}}{2},\varepsilon_{0}^{2}\right)-2\delta \left(N_{i^{\prime \prime }}+N_{j}-n_{i^{\prime \prime }}-n_{j},\frac{1}{2},\varepsilon_{0}\right). \end{array}$$

combining Equation (A26) and Equation (A27), we are clear that

(A28) $$\begin{array}{c} \left|n_{i}^{\prime \prime }-n_{j}\right|\leq \left(N_{i^{\prime \prime }}+N_{j}\right)\sqrt{1-F_{ij}^{2}}+2\delta \left(N_{i^{\prime \prime }}+N_{j},\frac{1+\sqrt{1-F_{ij}^{2}}}{2},\varepsilon_{0}^{2}\right)-2\delta \left(N_{i^{\prime \prime }}+N_{j}-n_{i^{\prime \prime }}-n_{j},\frac{1}{2},\varepsilon_{0}\right) \end{array}$$

holds with a failure probability $2\varepsilon_{0}$. For simplicity’s sake, we enlarge the R.H.S of Equation (A28). Concretely, we replace $2\delta (N_{i^{\prime \prime }}+N_{j}-n_{i^{\prime \prime }}-n_{j},\frac{1}{2},\varepsilon_{0}^{2})$ by $2\delta (N_{i^{\prime \prime }}+N_{j}-n_{i}-n_{j},\frac{1}{2},\varepsilon_{0}^{2})$ in Equation (A28), say

(A29) $$\begin{array}{c} |n_{i^{\prime \prime }}-n_{j}|\leq \left(N_{i^{\prime \prime }}+N_{j}\right)\sqrt{1-F_{ij}^{2}}+2\delta \left(N_{i^{\prime \prime }}+N_{j},\frac{1+\sqrt{1-F_{ij}^{2}}}{2},\varepsilon_{0}^{2}\right)-2\delta \left(N_{i^{\prime \prime }}+N_{j}-n_{i}-n_{j},\frac{1}{2},\varepsilon_{0}\right). \end{array}$$

note that these bounds of statistical fluctuations can be derived by the Chernoff bound [52]. Similarly, because of the probability that Alice sends the quantum state $\frac{1}{\sqrt{2}}{(|i^{{}^{\prime \prime }}\rangle }_{A}|\phi_{i}\rangle_{B}+{|j\rangle }_{A}|\phi_{j}\rangle_{B})$ is $2P_{j}$, using the well-known Chernoff bound [52], we know that

(A30) $$\begin{array}{c} 2N_{tot}P_{j}-\delta \left(N_{tot},2P_{j},\varepsilon_{1}\right)\leq N_{i^{\prime \prime }}+N_{j}\leq 2N_{tot}P_{j}+\delta \left(N_{tot},2P_{j},\varepsilon_{1}\right) \end{array}$$

Combing Equation (A29) and Equation (A30), we know that

(A31) $$\begin{array}{c} \left|n_{i^{\prime \prime }}-n_{j}\right|\leq N_{1}\sqrt{1-F_{ij}^{2}}+2\delta \left(N_{1},\frac{1+\sqrt{1-F_{ij}^{2}}}{2},\varepsilon_{0}^{2}\right)-2\delta \left(N_{2}-n_{i}-n_{j},\frac{1}{2},\varepsilon_{0}\right) \end{array}$$

holds with a failure probability $2\varepsilon_{0}+2\varepsilon_{1}$, where $N_{1}=2N_{tot}P_{j}+\delta \left(N_{tot},2P_{j},\varepsilon_{1}\right)$ and $N_{2}=2N_{tot}P_{j}-\delta \left(N_{tot},2P_{j},\varepsilon_{1}\right)$ Finally, to derive the relation between $n_{i}$ and $n_{j}$, we have to consider the relations between $n_{i}$ and $n_{i^{\prime \prime }}$. It is easy to know that $n_{i^{\prime \prime }}=\frac{P_{j}}{P_{i}}n_{i}$ on average since there is no way for Eve to distinguish $|i^{\prime }\rangle$ and $|i^{\prime \prime }\rangle$. Hence, using the Chernoff bound [52] again, we know that

(A32) $$\begin{array}{c} \frac{P_{j}}{P_{i}}n_{i}-\delta \left(n_{i},\frac{P_{j}}{P_{i}},\varepsilon_{2}\right)\leq n_{i^{\prime \prime }}\leq \frac{P_{j}}{P_{i}}n_{i}+\delta \left(n_{i},\frac{P_{j}}{P_{i}},\varepsilon_{2}\right) \end{array}$$

combining Equation (A31) and Equation (A32), we can obtain the inequality Equation (A21). In conclusion, we complete the proof. □

With Lemma A1, we can derive the constrains between $n_{i|2\mu }$ and $n_{j|2\nu }$. Since Alice and Bob send the quantum state $\tau_{j|2\mu }$ ($\tau_{j|2\nu }$ ) with the probability $\frac{2P_{\mu }^{2}}{M}{\tilde{P}}_{j|2\mu }$($\frac{2P_{\nu }^{2}}{M}{\tilde{P}}_{j|2\nu }$) and the fidelity between them is $F_{\mu \nu }^{j}=\sum_{n=0}^{\infty }\frac{\sqrt{P_{j+Mn|2\mu }}\sqrt{P_{j+Mn|2\nu }}}{\sqrt{{\tilde{P}}_{j|2\mu }{\tilde{P}}_{j|2\nu }}}$. By Lemma A1, we can obtain the relation between $n_{j|2\mu }$ and $n_{j|2\nu }$ which reads

(A33) $$\begin{array}{c} |C_{j|2\mu }n_{j|2\mu }-C_{j|2\nu }n_{j|2\nu }|\leq {▵}_{\mu \nu }^{j}. \end{array}$$

We let $P_{1}=\frac{2P_{\mu }^{2}}{M}{\tilde{P}}_{j|2\mu }$ and $P_{2}=\frac{2P_{\nu }^{2}}{M}{\tilde{P}}_{j|2\nu }$. If $P_{1}>P_{2}$, we have $C_{j|2\mu }=\frac{P_{2}}{P_{1}}$,$C_{j|2\nu }=1$ and

(A34) $$\begin{array}{c} {▵}_{\mu \nu }^{j}=N_{1}\sqrt{1-{\left(F_{\mu \nu }^{j}\right)}^{2}}+2\delta \left(N_{1},\frac{1+\sqrt{1-{\left(F_{\mu \nu }^{j}\right)}^{2}}}{2},\varepsilon_{0}^{2}\right)-2\delta \left(N_{2}-n_{j|2\mu }-n_{j|2\nu },\frac{1}{2},\varepsilon_{0}\right)+\delta \left(n_{j|2\mu },\frac{P_{2}}{P_{1}},\varepsilon_{2}\right) \end{array}$$

where $N_{1}=2N_{tot}P_{2}+\delta \left(N_{tot},2P_{2},\varepsilon_{1}\right)$ and $N_{2}=2N_{tot}P_{2}-\delta \left(N_{tot},2P_{2},\varepsilon_{1}\right)$

If $P_{1}<P_{2}$, we have that $C_{j|2\nu }=\frac{P_{1}}{P_{2}}$,$C_{j|2\mu }=1$ and

(A35) $$\begin{array}{c} {▵}_{\mu \nu }^{j}=N_{1}\sqrt{1-{\left(F_{\mu \nu }^{j}\right)}^{2}}+2\delta \left(N_{1},\frac{1+\sqrt{1-{\left(F_{\mu \nu }^{j}\right)}^{2}}}{2},\varepsilon_{0}^{2}\right)-2\delta \left(N_{2}-n_{j|2\mu }-n_{j|2\nu },\frac{1}{2},\varepsilon_{0}\right)+\delta \left(n_{j|2\nu },\frac{P_{1}}{P_{2}},\varepsilon_{2}\right) \end{array}$$

where $N_{1}=2N_{tot}P_{1}+\delta \left(N_{tot},2P_{1},\varepsilon_{1}\right)$ and $N_{2}=2N_{tot}P_{1}-\delta \left(N_{tot},2P_{1},\varepsilon_{1}\right)$

One can know that the constraints Equations (A34) and (A35) are nonlinear because of the $n_{j|2\mu }$ in $\delta (n_{j|2\mu },\frac{P_{2}}{P_{1}},\varepsilon_{2})$ or $n_{j|2\nu }$ in $\delta (n_{j|2\nu },\frac{P_{1}}{P_{2}},\varepsilon_{2})$. To keep the linearity of these constraints for ease of numerical calculations, we replace $n_{j|2\mu }$ with $n_{2\mu }$ and replace $n_{j|2\nu }$ with $n_{2\nu }$ in ${▵}_{\mu \nu }^{j}$. That is to say, without compromising the security, we replace Equation (A34) by

(A36) $$\begin{array}{c} {▵}_{\mu \nu }^{j}=N_{1}\sqrt{1-{\left(F_{\mu \nu }^{j}\right)}^{2}}+2\delta \left(N_{1},\frac{1+\sqrt{1-{\left(F_{\mu \nu }^{j}\right)}^{2}}}{2},\varepsilon_{0}^{2}\right)-2\delta \left(N_{2}-n_{2\mu }-n_{2\nu },\frac{1}{2},\varepsilon_{0}\right)+\delta \left(n_{2\mu },\frac{P_{2}}{P_{1}},\varepsilon_{2}\right) \end{array}$$

and replace Equation (A35) by

(A37) $$\begin{array}{c} {▵}_{\mu \nu }^{j}=N_{1}\sqrt{1-{\left(F_{\mu \nu }^{j}\right)}^{2}}+2\delta \left(N_{1},\frac{1+\sqrt{1-{\left(F_{\mu \nu }^{j}\right)}^{2}}}{2},\varepsilon_{0}^{2}\right)-2\delta \left(N_{2}-n_{2\mu }-n_{2\nu },\frac{1}{2},\varepsilon_{0}\right)+\delta \left(n_{2\nu },\frac{P_{1}}{P_{2}},\varepsilon_{2}\right) \end{array}$$

moreover, recalling Alice and Bob may both choose intensity 0 and obtain the corresponding number of successful clicks $n_{0}$, we have two additional constraints between $n_{0}$ and $n_{0|2\mu },n_{0|2\nu }$ which reads

(A38) $$\begin{array}{c} |C_{0,\mu }n_{0}-C_{2\mu }^{0}n_{0|2\mu }|\leq {▵}_{0\mu }^{0}\\ |C_{0,\nu }n_{0}-C_{2\nu }^{0}n_{0|2\nu }|\leq {▵}_{0\nu }^{0} \end{array}$$

since the probability of both Alice and Bob sending the quantum state |0〉〈0| is $P_{O}^{2}$ and the fidelity between |0〉〈0| and $\frac{1}{2}|{\tilde{0}}_{2\mu }\rangle \langle {\tilde{0}}_{2\mu }|+\frac{1}{2}|{\tilde{0}}_{2\mu }\rangle^{\prime }\langle {\tilde{0}}_{2\mu }|$ ($\frac{1}{2}|{\tilde{0}}_{2\nu }\rangle \langle {\tilde{0}}_{2\nu }|+\frac{1}{2}|{\tilde{0}}_{2\nu }\rangle^{\prime }\langle {\tilde{0}}_{2\nu }|$ ) is $F_{\mu 0}^{0}=\frac{P_{0|2\mu }}{{\tilde{P}}_{0|2\mu }}$($F_{\nu 0}^{0}=\frac{P_{0|2\nu }}{{\tilde{P}}_{0|2\nu }}$), we can obtain the coefficients of these two constraints according to Lemma A1. For simplicity’s sake’s sake, we let $P_{1}=P_{O}^{2}$ and $P_{2}=\frac{2P_{\mu }^{2}{\tilde{P}}_{0|2\mu }}{M}$; then, if $P_{1}>P_{2}$, we have $C_{0,\mu }=\frac{P_{2}}{P_{1}},C_{2\mu }^{0}=1$ and

$$\begin{array}{c} {▵}_{0\mu }^{0}=N_{1}\sqrt{1-{\left(F_{\mu 0}^{0}\right)}^{2}}+2\delta \left(N_{1},\frac{1+\sqrt{1-{\left(F_{\mu 0}^{0}\right)}^{2}}}{2},\varepsilon_{0}^{2}\right)-2\delta \left(N_{2}-n_{2\mu }-n_{0},\frac{1}{2},\varepsilon_{0}\right)+\delta \left(n_{0},\frac{P_{2}}{P_{1}},\varepsilon_{2}\right) \end{array}$$

where $N_{1}=2N_{tot}P_{2}+\delta \left(N_{tot},2P_{2},\varepsilon_{1}\right)$ and $N_{2}=2N_{tot}P_{2}-\delta \left(N_{tot},2P_{2},\varepsilon_{1}\right)$; otherwise, we have $C_{0,\mu }=1,C_{2\mu }^{0}=\frac{P_{1}}{P_{2}}$ and $C_{0,\mu }=\frac{P_{2}}{P_{1}},C_{2\mu }^{0}=1$ and

(A39) $$\begin{array}{c} {▵}_{0\mu }^{0}=N_{1}\sqrt{1-{\left(F_{\mu 0}^{0}\right)}^{2}}+2\delta \left(N_{1},\frac{1+\sqrt{1-{\left(F_{\mu 0}^{0}\right)}^{2}}}{2},\varepsilon_{0}^{2}\right)-2\delta \left(N_{2}-n_{2\mu }-n_{0},\frac{1}{2},\varepsilon_{0}\right)+\delta \left(n_{2\mu },\frac{P_{1}}{P_{2}},\varepsilon_{2}\right) \end{array}$$

where $N_{1}=2N_{tot}P_{1}+\delta \left(N_{tot},2P_{1},\varepsilon_{1}\right)$ and $N_{2}=2N_{tot}P_{1}-\delta \left(N_{tot},2P_{1},\varepsilon_{1}\right)$.

Similarly, we let $P_{1}=P_{O}^{2}$ and $P_{2}=\frac{2P_{\nu }^{2}{\tilde{P}}_{0|2\nu }}{M}$; then, if $P_{1}>P_{2}$, we have $C_{0,\nu }=\frac{P_{2}}{P_{1}},C_{2\nu }^{0}=1$ and

$$\begin{array}{c} {▵}_{0\nu }^{0}=N_{1}\sqrt{1-{\left(F_{\nu 0}^{0}\right)}^{2}}+2\delta \left(N_{1},\frac{1+\sqrt{1-{\left(F_{\nu 0}^{0}\right)}^{2}}}{2},\varepsilon_{0}^{2}\right)-2\delta \left(N_{2}-n_{2\nu }-n_{0},\frac{1}{2},\varepsilon_{0}\right)+\delta \left(n_{0},\frac{P_{2}}{P_{1}},\varepsilon_{2}\right) \end{array}$$

where $N_{1}=2N_{tot}P_{2}+\delta \left(N_{tot},2P_{2},\varepsilon_{1}\right)$ and $N_{2}=2N_{tot}P_{2}-\delta \left(N_{tot},2P_{2},\varepsilon_{1}\right)$; otherwise, we have $C_{0,\nu }=1,C_{2\nu }^{0}=\frac{P_{1}}{P_{2}}$ and $C_{0,\nu }=\frac{P_{2}}{P_{1}},C_{2\nu }^{0}=1$ and

(A40) $$\begin{array}{c} {▵}_{0\nu }^{0}=N_{1}\sqrt{1-{\left(F_{\nu 0}^{0}\right)}^{2}}+2\delta \left(N_{1},\frac{1+\sqrt{1-{\left(F_{\nu 0}^{0}\right)}^{2}}}{2},\varepsilon_{0}^{2}\right)-2\delta \left(N_{2}-n_{2\nu }-n_{0},\frac{1}{2},\varepsilon_{0}\right)+\delta \left(n_{2\nu },\frac{P_{1}}{P_{2}},\varepsilon_{2}\right) \end{array}$$

where $N_{1}=2N_{tot}P_{1}+\delta \left(N_{tot},2P_{1},\varepsilon_{1}\right)$ and $N_{2}=2N_{tot}P_{1}-\delta \left(N_{tot},2P_{1},\varepsilon_{1}\right)$.

Now the gaps between $n_{j|2\mu }$ v.s. $n_{j|2\nu }$, $n_{0|2\mu }$ v.s. $n_{0}$, and $n_{0|2\nu }$ v.s. $n_{0}$ have been given. To bound $n_{ph}$, we can now resort to linear programming below:

(A41) $$\begin{array}{cc} \max\; & n_{ph}=\sum_{j=0,j\in N_{0}}^{M-2}n_{j|2\mu }\\ s.t.\; & \sum_{j=0}^{M-1}n_{j|2\mu }=n_{2\mu }\\ & \sum_{j=0}^{M-1}n_{j|2\nu }=n_{2\nu }\\ & |C_{0,\mu }n_{0}-C_{2\mu }^{0}n_{0|2\mu }|\leq {▵}_{0\mu }^{0}\\ & |C_{0,\nu }n_{0}-C_{2\nu }^{0}n_{0|2\nu }|\leq {▵}_{0\nu }^{0}\\ & |C_{j|2\mu }n_{j|2\mu }-C_{j|2\nu }n_{j|2\nu }|\leq {▵}_{\mu \nu }^{j}. \end{array}$$

For ease of calculation, in all these constraints we let $\varepsilon_{0}=\varepsilon_{1}=\varepsilon_{2}=\varepsilon_{a}$; then, the total failure probability that we obtain the bound ${▵}_{\mu \nu }^{j}$ is $6\varepsilon_{a}$. Meanwhile, the failure probability that we obtain the last two bounds on is $2\varepsilon_{a}$. Hence, the total failure probability of all these constraints is $6\left(M+2\right)\varepsilon_{a}$. To conclude, with the help of the linear programming in Equation (A41), one can calculate the upper-bound of $n_{ph}$.

For simplicity’s sake, we give the analytical solution of this linear programming below. We divide $n_{ph}$ into two parts, say

(A42) $$\begin{array}{c} n_{ph}=n_{0|2\mu }+\sum_{j=2,4,6}n_{j|2\mu }. \end{array}$$

according to the inequality $|C_{0,\mu }n_{0}-C_{2\mu }^{0}n_{0|2\mu }|\leq {▵}_{0\mu }^{0}$, we can obtain that the bounds of $n_{0|2\mu }$ are

(A43) $$\begin{array}{c} \bar{n_{0|2\mu }}=\frac{C_{0,\mu }n_{0}+{▵}_{0\mu }^{0}}{C_{2\mu }^{0}}, \end{array}$$

(A44) $$\begin{array}{c} \underline{n_{0|2\mu }}=\frac{C_{0,\mu }n_{0}-{▵}_{0\mu }^{0}}{C_{2\mu }^{0}}. \end{array}$$

similarly, with the inequality $|C_{0,\nu }n_{0}-C_{2\nu }^{0}n_{0|2\nu }|\leq {▵}_{0\nu }^{0}$, we have the bounds of $n_{0|2\nu }$, i.e.,

(A45) $$\begin{array}{c} \bar{n_{0|2\nu }}=\frac{C_{0,\nu }n_{0}+{▵}_{0\nu }^{0}}{C_{2\nu }^{0}}, \end{array}$$

(A46) $$\begin{array}{c} \underline{n_{0|2\nu }}=\frac{C_{0,\nu }n_{0}-{▵}_{0\nu }^{0}}{C_{2\nu }^{0}}. \end{array}$$

besides the bound $\bar{n_{0|2\mu }}$, we need to calculate the upper bound of $\sum_{j=2,4,6}n_{j|2\mu }$ which we derive below. If we set μ>ν and $P_{\mu }>P_{\nu }$, according to Equation A33, we have

(A47) $$\begin{array}{cc} C_{j|2\mu }= & \frac{P_{\nu }^{2}{\tilde{P}}_{j|2\nu }}{P_{\mu }^{2}{\tilde{P}}_{j|2\mu }},\\ C_{j|2\nu }= & 1, \end{array}$$

for j≥1. Hence, $n_{j|2\nu }\leq \frac{P_{\nu }^{2}{\tilde{P}}_{j|2\nu }}{P_{\mu }^{2}{\tilde{P}}_{j|2\mu }}n_{j|2\mu }+{▵}_{\mu \nu }^{j}$ where j≥1 hold. Then, combing with the equality $n_{2\nu }=\sum_{j=0}^{M}n_{j|2\nu }$, we have

(A48) $$\begin{array}{c} \sum_{j=1,3,5,7}\frac{P_{\nu }^{2}{\tilde{P}}_{j|2\nu }}{P_{\mu }^{2}{\tilde{P}}_{j|2\mu }}n_{j|2\mu }+\sum_{j=2,4,6}\frac{P_{\nu }^{2}{\tilde{P}}_{j|2\nu }}{P_{\mu }^{2}{\tilde{P}}_{j|2\mu }}n_{j|2\mu }\geq n_{2\nu }-\sum_{j=1}^{7}{▵}_{\mu \nu }^{j}-\bar{n_{0|2\nu }}. \end{array}$$

It is easy to prove that

(A49) $$\begin{array}{c} \frac{P_{\nu }^{2}{\tilde{P}}_{j|2\nu }}{P_{\mu }^{2}{\tilde{P}}_{j|2\mu }}\geq \frac{P_{\nu }^{2}{\tilde{P}}_{j+1|2\nu }}{P_{\mu }^{2}{\tilde{P}}_{j+1|2\mu }} \end{array}$$

for j≥1 holds in the case of μ>ν and $P_{\mu }>P_{\nu }$. Consequently, we can know that

(A50) $$\begin{array}{c} \frac{P_{\nu }^{2}{\tilde{P}}_{1|2\nu }}{P_{\mu }^{2}{\tilde{P}}_{1|2\mu }}\left(\sum_{j=1,3,5,7}n_{j|2\mu }\right)+\frac{P_{\nu }^{2}{\tilde{P}}_{2|2\nu }}{P_{\mu }^{2}{\tilde{P}}_{2|2\mu }}\left(\sum_{j=2,4,6}n_{j|2\mu }\right)\geq n_{2\nu }-\sum_{j=1}^{7}{▵}_{\mu \nu }^{j}-\bar{n_{0|2\nu }}. \end{array}$$

Finally, combing with the equality $\sum_{j=0}^{M-1}n_{j|2\mu }=n_{2\mu }$, Equations (A43) and (A45), we obtain the upper bound given by

(A51) $$\begin{array}{c} \sum_{j=2,4,6}n_{j|2\mu }\leq \frac{\frac{P_{\nu }^{2}{\tilde{P}}_{1|2\nu }}{P_{\mu }^{2}{\tilde{P}}_{1|2\mu }}n_{2\mu }-n_{2\nu }+\sum_{j=1}^{7}{▵}_{\mu \nu }^{j}+\frac{C_{0,\nu }n_{0}+{▵}_{0\nu }^{0}}{C_{2\nu }^{0}}-\frac{C_{0,\mu }n_{0}-{▵}_{0\mu }^{0}}{C_{2\mu }^{0}}\frac{P_{\nu }^{2}{\tilde{P}}_{1|2\nu }}{P_{\mu }^{2}{\tilde{P}}_{1|2\mu }}}{\frac{P_{\nu }^{2}{\tilde{P}}_{1|2\nu }}{P_{\mu }^{2}{\tilde{P}}_{1|2\mu }}-\frac{P_{\nu }^{2}{\tilde{P}}_{2|2\nu }}{P_{\mu }^{2}{\tilde{P}}_{2|2\mu }}}. \end{array}$$

Finally, we have the upper bound of $n_{ph}$, say

(A52) $$\begin{array}{c} n_{ph}^{U}=\frac{\frac{P_{\nu }^{2}{\tilde{P}}_{1|2\nu }}{P_{\mu }^{2}{\tilde{P}}_{1|2\mu }}n_{2\mu }-n_{2\nu }+\sum_{j=1}^{7}{▵}_{\mu \nu }^{j}+\frac{C_{0,\nu }n_{0}+{▵}_{0\nu }^{0}}{C_{2\nu }^{0}}-\frac{C_{0,\mu }n_{0}-{▵}_{0\mu }^{0}}{C_{2\mu }^{0}}\frac{P_{\nu }^{2}{\tilde{P}}_{1|2\nu }}{P_{\mu }^{2}{\tilde{P}}_{1|2\mu }}}{\frac{P_{\nu }^{2}{\tilde{P}}_{1|2\nu }}{P_{\mu }^{2}{\tilde{P}}_{1|2\mu }}-\frac{P_{\nu }^{2}{\tilde{P}}_{2|2\nu }}{P_{\mu }^{2}{\tilde{P}}_{2|2\mu }}}+\frac{C_{0,\mu }n_{0}+{▵}_{0\mu }^{0}}{C_{2\mu }^{0}}. \end{array}$$
